# Associations between pathologic tumor features and preadjuvant therapy cognitive performance in women diagnosed with breast cancer

**DOI:** 10.1002/cam4.964

**Published:** 2017-01-13

**Authors:** Theresa A. Koleck, Catherine M. Bender, Susan M. Sereika, Christopher M. Ryan, Puja Ghotkar, Adam M. Brufsky, Rachel C. Jankowitz, Priscilla F. McAuliffe, Beth Z. Clark, Yvette P. Conley

**Affiliations:** ^1^ University of Pittsburgh School of NursingPittsburghPennsylvania; ^2^ Columbia University School of NursingNew YorkNew York; ^3^ Departments of Biostatistics and EpidemiologyUniversity of Pittsburgh Graduate School of Public HealthPittsburghPennsylvania; ^4^ Department of PsychiatryUniversity of California San FranciscoSan FranciscoCalifornia; ^5^ Department of PsychiatryUniversity of PittsburghPittsburghPennsylvania; ^6^ Division of Hematology/OncologyMagee‐Womens Hospital of UPMCPittsburghPennsylvania; ^7^ University of Pittsburgh Cancer InstitutePittsburghPennsylvania; ^8^ University of Pittsburgh School of MedicinePittsburghPennsylvania; ^9^ Magee‐Womens Hospital of UPMCCancerCenterPittsburghPennsylvania; ^10^ Division of Breast Surgical OncologyMagee‐Womens Hospital of UPMCPittsburghPennsylvania; ^11^ Division of Gynecologic PathologyMagee‐Womens Hospital of UPMCPittsburghPennsylvania; ^12^ Department of Human GeneticsUniversity of Pittsburgh Graduate School of Public HealthPittsburghPennsylvania

**Keywords:** biomarkers, Breast neoplasms, cognition, pathology, receptor ErbB‐2

## Abstract

Intertumor heterogeneity has been proposed as a potential mechanism to account for variability in cognitive performance in women diagnosed with breast cancer. The purpose of this study was to explore associations between variation in pathologic tumor features (PTFs) and variability in preadjuvant therapy cognitive performance in postmenopausal women newly diagnosed with early‐stage breast cancer. Participants (*N* = 329) completed a comprehensive battery of neuropsychological tests to evaluate cognitive performance after primary surgery but prior to initiation of adjuvant anastrozole±chemotherapy. PTF data were abstracted from medical records. Robust multiple linear regression models were fit to estimate associations between individual PTFs and the cognitive function composite domain scores. All models controlled for age, estimated intelligence, and levels of depressive symptoms, anxiety, fatigue, and pain. Diagnosis of a HER2‐positive tumor contributed to poorer verbal (*b* = −0.287, *P* = 0.018), visual (*b* = −0.270, *P* = 0.001), and visual working (*b* = −0.490, *P* < 0.001) memory performance compared to diagnosis of a HER2‐negative tumor. Similarly, as HER2 immunohistochemistry classification score increased, verbal (*b* = −0.072, *P* = 0.093), visual (*b* = −0.081, *P* = 0.003), and visual working (*b* = −0.170, *P* < 0.001) memory performance score decreased. Associations with performance were also noted between location, focality/centricity, hormone receptor expression, cellular proliferation (i.e., Ki67), and Oncotype DX ^®^ Breast Cancer Assay Recurrence Score^®^.) Our results suggest that certain PTFs related to more aggressive tumor phenotypes or inferior breast cancer prognosis may be implicated in poorer preadjuvant therapy cognitive performance. Follow‐up studies that include a cognitive assessment before primary surgery should be conducted to further delineate the role of intertumor heterogeneity on cognitive performance.

## Introduction

Current studies suggest that approximately 20–40% of women newly diagnosed with breast cancer experience lower than expected cognitive performance prior to receiving adjuvant breast cancer therapy 1, 2, 3, 4. Findings that only a subgroup of women diagnosed with breast cancer are vulnerable to preadjuvant therapy cognitive dysfunction have led to investigations focused on factors that predispose certain women to these burdensome cognitive changes. As succinctly summarized in a recent review by Wefel, Kesler, Noll, and Schagen, preadjuvant therapy cognitive dysfunction has been found to be unrelated to distress, fatigue, comorbidities, or surgery‐related factors 5. Consequently, a number of proposed mechanisms have emerged to account for observed changes and variation in preadjuvant therapy cognitive performance including the biology of the cancer itself 5, 6, 7, 8. Breast carcinoma biology is heterogeneous and characterized by high degrees of molecular and pathologic diversity both among breast cancers diagnosed in different individuals (i.e., intertumor) and within the same individual (i.e., intratumor). The ability of intertumor pathologic tumor feature (PTF) heterogeneity to account for observed variability in cognitive performance among women diagnosed with breast cancer is a particularly intriguing hypothesis 9, 10, 11.

Evidence exists to support the investigation of PTFs and preadjuvant therapy cognitive dysfunction in women with breast cancer. In cohorts of women 18–70 (mean: 54.0) years of age inclusive of all stages of menopause, Ahles et al. reported that 22% of women newly diagnosed with invasive breast cancer (stage I, II, or IIIA) had lower than expected cognitive performance compared to 0% of women with noninvasive breast cancer (stage 0) and 4% healthy controls 4. They further report that the verbal ability, memory, and sorting domains most commonly contributed to a lower than expected cognitive performance classification 4. Additionally, women diagnosed with invasive breast cancer had significantly poorer reaction time compared to healthy controls; in contrast, women diagnosed with noninvasive breast cancer performed as well as healthy controls 4. Mandelblatt and colleagues found that older women (≥ 60 years of age) newly diagnosed with stage II to III nonmetastatic breast cancer had lower preadjuvant therapy executive function domain scores compared to participants with stage 0 or I breast cancer after adjustment for race, years of education, recruitment site, and type of surgery (i.e., mastectomy vs. lumpectomy) 12. Associations between stage (0 and I versus II and III) were not observed with the language, executive function, learning and memory, visual‐spatial, or attention, working memory, and processing speed language domains 12. Conflicting results have also been reported. Lange et al. found no associations between cancer stage, hormonal receptor status, or HER2 status and preadjuvant therapy cognitive deficits in elderly women (>65 years of age) newly diagnosed with breast cancer 3. Likewise, Wefel et al. did not report any differences in cognitive impairment between women (54 ± 9.1 years of age) diagnosed with stage I to II breast cancer and those diagnosed with stage IIIA breast cancer 2.

Although limited, these reported associations and contradictory findings warrant further investigation related to the impact of PTF variation on preadjuvant therapy cognitive performance. Additionally, studies to date have not comprehensively interrogated this hypothesis examining limited PTFs and focusing mainly on breast carcinoma stage. Therefore, the purpose of this exploratory, ancillary study is to investigate associations between variation in a more comprehensive set of PTFs routinely obtained to characterize a malignant breast tumor and included as part of a surgical pathology report and variability in cognitive performance in postmenopausal women newly diagnosed with early‐stage breast cancer after surgery but prior to initiation of adjuvant cancer therapies.

## Materials and Methods

### Participants

Participants were recruited from the Comprehensive Breast Cancer Program of the University of Pittsburgh Cancer Institute as part of the parent study, Anastrozole Use in Menopausal Women (R01CA107408). The available sample for this ancillary study was comprised of 369 postmenopausal women newly diagnosed with hormone receptor positive early‐stage (i.e., American Joint Committee on Cancer [AJCC] breast carcinoma stage I, IIA, IIB, or IIIA) breast cancer scheduled to receive adjuvant anastrozole ± chemotherapy at a future date. Sample sizes varied for individual PTFs based on availability from the medical record. In addition to being postmenopausal, all women were 75 years of age or younger, able to speak and read English, and completed at least 8 years of education. Participants with a history of cancer, neurologic disease, or recent (≤2 years) self‐reported hospitalization for psychiatric illness were excluded. Both the ancillary and parent studies were approved by the University of Pittsburgh Institutional Review Board. Written informed consent was obtained from all participants.

### Evaluation of cognitive function

Cognitive performance was assessed after surgery for tumor removal but prior to initiation of adjuvant cancer therapy. Participants completed a comprehensive battery of neuropsychological tests to evaluate performance related to eight cognitive function composite domains: attention (Cambridge Neuropsychological Test Automated Battery [CANTAB] Rapid Visual Information Processing Test);^13^ concentration (Digit Vigilance Test);^14^ executive function (CANTAB Stockings of Cambridge Test and CANTAB Spatial Working Memory Test);^13^ mental flexibility (Delis Kaplan Executive Function System Color‐Word Interference Test);^15^ psychomotor speed (Grooved Pegboard Test and Digit Symbol Substitution Test);^16^, ^17^ verbal memory (Rey Auditory Verbal Learning Test, Delis Kaplan Executive Function Verbal Fluency Test, and Rivermead Story Test);^15^, ^18^, ^19^ visual memory (CANTAB Paired Associates Learning Test and Rey Complex Figure Test);^13^, ^20^ and visual working memory (CANTAB Stockings of Cambridge Test and Rey Complex Figure Test) 13, 20. Cognitive performance is reported as a *Z*‐score with more negative scores indicating poorer performance. Details of the neuropsychological test battery, composite domains, and *Z*‐score calculation have been published previously 21. Age (in years), estimated verbal intelligence (National Adult Reading Test‐Revised) 22, depressive symptoms (Beck Depression Inventory‐II) 23, anxiety and fatigue (Profile of Mood States Tension‐Anxiety and Fatigue‐Inertia subscales, respectively) 24, and current pain at time of assessment (Brief Pain Inventory)^25^ were also evaluated.

### Evaluation of PTFs

PTF data were obtained from surgical pathology reports of study participants and included the following: AJCC breast carcinoma stage (I, IIA, IIB, or IIIA);^26^ tumor classification (T1a, T1b, T1c, T2, T3);^26^ lymph node status (positive or negative); number of positive lymph nodes; tumor laterality (left or right breast); tumor location within breast (clock position and/or reported quadrant location [upper outer, upper inner, lower outer, or lower inner]); tumor focality/centricity (single or multiple); primary tumor size (measured to the nearest millimeter); aggregate tumor size if multifocal/centric (measured to the nearest millimeter); histologic type (invasive ductal, invasive lobular, or both);^27^ Nottingham Score (score 3–9);^28^ Nottingham Grade (Grade 1 [low], Grade 2 [intermediate], or Grade 3 [high]);^28^ lymphovascular invasion (presence or absence); estrogen receptor (ER) status (positive or negative); ER H‐score (extent of nuclear immunoreactivity) quantitation (score 0–300); ER Oncotype DX^®^ Breast Cancer Assay quantitative single gene score (score 0–12.5 + ; Negative <6.5, Positive ≥6.5);^29^ progesterone receptor (PR) status (positive or negative); PR H‐score (extent of nuclear immunoreactivity) quantitation (score: 0–300); PR Oncotype DX^®^ Breast Cancer Assay quantitative single gene score (score 0–10 + ; Negative <5.5, Positive ≥5.5);^29^ HER2 immunohistochemistry (IHC) classification score (0, 1 +  [Negative], 2 +  [Equivocal], or 3 +  [Positive]);^30^ HER2/neu status (positive or negative based on IHC test and/or FISH amplification); HER2 Oncotype DX^®^ Breast Cancer Assay quantitative single gene score (score: 0–13 + ; Negative <10.7, Equivocal: 10.7–11.5, Positive ≥11.5);^29^ Ki67 index (0–100%; percentage of total number of tumor cells with nuclear staining); Ki67 proliferative rate classification (Low [≤10%], Moderate [11–25%], High [26–50%], or Very High [>50%]); and Oncotype DX^®^ Breast Cancer Assay Recurrence Score^®^ (score: 0–100 from multigene expression algorithm) 31, 32. In instances where a participant had more than one primary breast tumor in the same breast, multifocal/centric, or bilateral breast cancer, characteristics of the tumor/foci contributing to the highest breast carcinoma stage were used in analyses.

Clock position and reported breast quadrant were used to categorize tumor location into quadrants (upper outer, upper inner, lower outer, and lower inner) plus retroareolar. Tumors located at the junction of two quadrants were assigned to the adjacent clockwise quadrant in the left breast and the adjacent counterclockwise quadrant in the right breast (e.g., lower junction would be assigned to the lower inner quadrant). In order to allow for the unique characterization of tumors located at the junction of two quadrants, tumor location was further described using octants (upper outer, upper inner, lower outer, lower inner, upper junction [12 o'clock], lower junction [6 o'clock], outer junction [left breast‐3 o'clock; right breast‐9 o'clock], and inner junction [left breast‐9 o'clock; right breast‐3 o'clock]) and retroareolar.

As a supplement to Oncotype DX^®^ Breast Cancer Assay Recurrence Scores^®^, Magee Equation recurrence scores were calculated using the three equations described in Klein et al 33. The three equations, which produce very similar results, use different combinations of Nottingham Score, ER H‐score, PR H‐score, HER2 status (negative, equivocal, or positive), tumor size, and/or Ki67 index to estimate Oncotype DX^®^ Breast Cancer Assay Recurrence Scores^®^ and corresponding recurrence risk category assignment (i.e., low, intermediate, or high). Thus, up to three scores were calculated for each participant based on available information. Scores from the three equations were reduced into a single variable giving preference to generated scores in the following sequence: equation 1 > equation 2 > equation 3. Scores from equation 1 were prioritized as this equation was found to most accurately replicate extreme values (i.e., assignment into the low and high recurrence risk categories). If a score from equation 1 was not available for a participant, the score from equation 2 was selected due to its concordance with Oncotype DX^®^ Breast Cancer Assay risk category overall and comparable performance to equation 3 when the intermediate risk category was omitted.

Abstracted PTF data were independently entered into a computer database by two individuals and compared for discrepancies. Discrepancies were adjudicated via independent review of raw data by a third individual. Furthermore, detailed data screening procedures were performed to ensure data accuracy. Data from individual PTFs were cross‐checked with other, directly corresponding PTFs (e.g., tumor classification and tumor size). Inconsistencies were addressed by reviewing raw data from PTF abstraction forms and/or the original pathology reports.

### Statistical analysis

Data were screened for anomalies prior to analyses. Standard descriptive statistics were computed for all variables. Wilcoxon–Mann–Whitney tests were used to compare the medians of cognitive function composite scores and covariates/confounders of participants included in the analysis to those excluded due to incomplete cognitive function, covariate/confounder, or PTF data. To adjust for potential influential points and heteroscedasticity, robust multiple linear regression models using Huber weighting and biweighting iterations were fit to estimate associations between individual PTFs and the cognitive function composite domain scores. All models controlled for age, estimated intelligence, and levels of depressive symptoms, anxiety, fatigue, and pain. Underlying assumptions were assessed for each regression model, including normality, linearity, homoscedasticity, and multicollinearity. In order to identify potentially influential points, Cook's distance was generated and evaluated as part of jackknifed residual by predicted value scatterplots. Due to the exploratory nature of this analysis, unstandardized regression coefficients and tests at a two‐tailed significance level of 0.05 were used to designate statistical significance. Statistical analyses were performed using Stata^®^ Data Analysis and Statistical Software SE Version 14.1 (StataCorp, College Station, TX) and IBM^®^ SPSS^®^ Statistics Version 23 (IBM Corp., Armonk, NY).

## Results

### Participant and breast cancer tumor characteristics

Of the 369 women diagnosed with early‐stage breast cancer enrolled in the parent study, 329 participants had PTF data collected and complete confounder/covariate information and cognitive function scores available for one or more cognitive function composite domains (Table 1). In general, participants were an average of 61.05 years of age, well‐educated (mean of 14.80 years of education), married or currently living with a partner (67.8%), and Caucasian (96.4%). A comparison of characteristics of participants included (*n* = 329) to those not included because PTF or pretreatment cognitive function data were not available or covariate/confounder information was incomplete (*n* = 40) revealed that participants not included in the analysis had poorer (*P* = 0.011) median attention performance *Z*‐scores (25% = −1.11, 50% = −0.43, 75% = 0.11) than participants included in the analysis (25% = −0.66, 50% = −0.12, 75% = 0.51).

**Table 1:** Participant Characteristics (*N* = 329)

Characteristic (Measure)	Mean±SD, Median or *n* (%)	Minimum	Maximum
Age (years)	61.05 ± 5.976, 61	45	75
Education (years)	14.80 ± 2.805, 14	6	26
Estimated verbal intelligence (NART‐R)	108.45 ± 8.584, 110.01	77.08	125.14
Depressive symptoms (BDI‐II)	5.33 ± 5.619, 4	0	32
Anxiety (POMS Tension‐Anxiety subscale)	7.64 ± 5.801, 7	0	29
Fatigue (POMS Fatigue‐Inertia subscale)	5.72 ± 5.986, 4	0	27
Pain (BPI)	1.44 ± 2.165, 0	0	9
Marital Status, currently married or living with significant other	223 (67.8)	–	–
Number of Children	1.89 ± 1.237, 2	0	7
Race, Caucasian	317 (96.4)	–	–
Cognitive function composite *Z*‐scores			
Attention, *n* = 321	−0.1587 ± 0.93945, −0.1243	−4.25	1.63
Concentration, *n* = 328	−0.0141 ± 0.91255, −0.1069	−3.41	3.98
Executive function, *n* = 329	−0.3953 ± 0.63810, −0.4290	−2.37	1.83
Mental flexibility, *n* = 328	0.1197 ± 0.78899, 0.2585	−4.05	1.63
Psychomotor speed, *n* = 329	−0.1201 ± 0.92513, 0.0149	−6.01	2.28
Verbal memory, *n* = 329	−0.2088 ± 0.66864, −0.1969	−2.58	1.28
Visual memory, *n* = 329	0.0680 ± 0.66866, 0.2600	−3.28	0.86
Visual working memory, *n* = 329	−0.0035 ± 0.78009, 0.1235	−4.73	1.55

BDI‐II, Beck Depression Inventory‐II; BPI, Brief Pain Inventory; PTF, pathologic tumor feature; NART‐R, National Adult Reading Test‐revised; POMS, Profile of Mood States; SD, standard deviation. Only participants with complete confounder/covariate information are included in the participant characteristic statistics.

The majority of breast cancer tumors were ductal (86.9%), single focus (84.2%), breast carcinoma stage I (65%), tumor classification T1c (40.4%), lymph node negative (77.5%), ER positive (98.8%), PR positive (87.8%), and HER2 negative (91.2%). The mean Nottingham Score (6.04 ± 1.306) for all tumors included in the analysis corresponds to an intermediate Nottingham Grade, and the mean Ki67 index (23.10 ± 21.522) reflects a moderate Ki67 classification. Oncotype DX^®^ Breast Cancer Assay Recurrence Scores^®^ ranged from 0 to 63 with a mean score of 18.26 ± 9.76. Similarly, Magee Equation recurrence scores ranged from 1.92 to 48.87 with a mean score of 20.51 ± 7.77. A summary of PTF data is reported in Table 2.

**Table 2:** PTF summary statistics (*N* = 329)

Feature	Mean±SD, Median or *n* (%)	Minimum	Maximum
AJCC tumor stage, *n* = 329			
Stage I	214 (65)	—	—
Stage IIA	75 (22.8)	—	—
Stage IIB	24 (7.3)	—	—
Stage IIIA	16 (4.9)	—	—
Tumor size (cm), *n* = 328	1.66 ± 1.500, 1.3	0.10	14.00
Aggregate tumor size (cm), *n* = 328	1.80 ± 1.599, 1.4	0.10	14.00
Tumor classification, *n* = 329			
T1a	37 (11.2)	—	—
T1b	82 (24.9)	—	—
T1c	133 (40.4)	—	—
T2	65 (19.8)	—	—
T3	12 (3.6)	—	—
Lymph node, *n* = 325			
Positive	73 (22.5)	—	—
Negative	252 (77.5)	—	—
Number of positive nodes, *n* = 329	0.42 ± 1.054, 0	0	8
Tumor focality/centricity, *n* = 329			
Single	277 (84.2)	—	—
Multiple	52 (15.8)	—	—
Tumor laterality, *n* = 329			
Right breast	149 (45.3)	—	—
Left breast	180 (54.7)	—	—
Tumor location octant, *n* = 323			
Upper outer	125 (38.7)	—	—
Lower outer	28 (8.7)	—	—
Lower inner	21 (6.5)	—	—
Upper inner	42 (13.0)	—	—
Upper junction	38 (11.8)	—	—
Lower junction	17 (5.3)	—	—
Outer junction	30 (9.3)	—	—
Inner junction	9 (2.8)	—	—
Retroareolar	13 (4.0)	—	—
Tumor location quadrant, *n* = 323			
Upper outer	163 (50.5)	—	—
Lower outer	58 (18.0)	—	—
Lower inner	38 (11.8)	—	—
Upper inner	51 (15.8)	—	—
Retroareolar	13 (4.0)	—	—
Invasive type, *n* = 329			
Ductal	285 (86.9)	—	—
Lobular	35 (10.7)	—	—
Ductal & lobular	8 (2.4)	—	—
Nottingham score, *n* = 315	6.04 ± 1.306, 6	3	9
Nottingham grade, *n* = 316			
Grade 1	95 (30.1)	—	—
Grade 2	171 (54.1)	—	—
Grade 3	50 (15.8)	—	—
ER Status, *n* = 328			
Positive	324 (98.8)	—	—
Negative	4 (1.2)	—	—
ER H‐score, *n* = 311	256.90 ± 59.978, 280	0	300
Oncotype DX ER score, *n* = 102	10.287 ± 1.056, 10.45	7.8	12.5
PR Status, *n* = 328			
Positive	288 (87.8)	—	—
Negative	40 (12.2)	—	—
PR H‐score, *n* = 310	130.08 ± 101.301, 130	0	300
Oncotype DX PR score, *n* = 102	7.08 ± 1.569, 7.20	3.2	10.2
HER2 status, *n* = 318			
Positive	28 (8.8)	—	—
Negative	290 (91.2)	—	—
HER2 IHC score, *n* = 291	1.21 ± 0.869, 1	0	3
Oncotype DX HER2 score, *n* = 74	8.93 ± 0.812, 8.90	7.6	12.8
LV invasion, *n* = 323			
Present	68 (21.1)	—	—
Absent	255 (78.9)	—	—
Ki67 classification, *n* = 169			
Low	66 (39.1)	—	—
Moderate	50 (29.6)	—	—
High	34 (20.1)	—	—
Very High	19 (11.2)	—	—
Ki67 Index, *n* = 168	23.10 ± 21.522, 15	1	90
Oncotype DX Recurrence Score^®^, *n* = 160	18.26 ± 9.76, 18	0	63
Magee equation recurrence score, *n* = 298	20.51 ± 7.77, 18.89	1.92	48.87

AJCC, American Joint Committee on Cancer; ER, estrogen receptor; HER2, human epidermal growth factor receptor 2; IHC, immunohistochemistry; LV, lymphovascular; Oncotype DX, Genomic Health Inc. Oncotype DX^®^ Breast Cancer Assay; PR, progesterone receptor; PTF, pathologic tumor feature; SD, standard deviation; TNM, Tumor, Node, Metastasis Classification of Malignant Tumors. Only participants with complete confounder/covariate information are included in the summary statistics.

### PTFs and preadjuvant therapy cognitive performance

Regression coefficients and *P* ‐values from all robust regression models evaluating the relation between PTFs and preadjuvant therapy cognitive performance are reported in Table S1. The most significant findings were related to memory and HER2 status and HER2 IHC classification score. Diagnosis of a HER2‐positive tumor contributed to poorer verbal (*b* = −0.287, *P* = 0.018), visual (*b* = −0.270, *P* = 0.001), and visual working (*b* = −0.490, *P* < 0.001) memory performance compared to diagnosis of a HER2‐negative tumor. Likewise, as HER2 IHC classification scores increased, verbal (*b* = −0.072, *P* = 0.093), visual (*b* = −0.081, *P* = 0.003), and visual working (*b* = −0.170, *P* < 0.001) memory performance scores decreased. In addition to associations with HER2 status and HER2 IHC score, a significant association was noted between tumor focality/centricity and verbal memory (*b* = −0.278, *P* = 0.003), such that possession of a multifocal/centric tumor contributed to poorer performance compared to a single focus tumor. While not statistically significant, this trend was observed across all domains. Diagnosis of a progesterone receptor (PR) positive tumor, compared to a PR‐negative tumor, also contributed to poorer verbal memory performance (*b* = −0.256, *P* = 0.015).

Tumor location effects were also noted. Diagnosis of a tumor located in the left breast, compared to the right breast, contributed positively to verbal memory (*b* = 0.156, *P* = 0.025) and visual working memory (*b* = 0.163, *P* = 0.026) performance scores. Overall tumor location quadrant was found to be significantly (*P* = 0.018) related to visual working memory. Specifically, having been diagnosed with a tumor in the lower inner quadrant contributed to poorer visual working performance compared to having been diagnosed a tumor in the upper outer quadrant (*b* = −0.267, *P* = 0.025). This same trend is observed with the lower inner octant location designation. A statistically significant association is also noted between lower junction octant, compared to upper outer octant, and visual working memory performance (*b* = −0.387, *P* = 0.023). Although tumor location did not significantly contribute to the model as a whole, diagnosis of a tumor in the lower inner quadrant or octant contributed to poorer mental flexibility performance compared to diagnosis of a tumor in the upper outer quadrant or octant (*b* = −0.335, *P* = 0.005; *b* = −0.441, *P* = 0.005) and diagnosis of a retroareolar tumor contributed to better visual memory performance compared to diagnosis of a tumor in the upper outer quadrant or octant (*b* = 0.259, *P* = 0.028; *b* = 0.228, *P* = 0.017).

Additionally, as Oncotype DX^®^ Breast Cancer Assay Recurrence Scores^®^ increased, mental flexibility performance scores decreased (*b* = −0.010, *P* = 0.032). Finally, Ki67 classification was found to be significantly associated with concentration performance (*P* = 0.042). In particular, a moderate Ki67 classification contributed positively to cognitive function performance compared to a low Ki67 classification (*b* = 0.381, *P* = 0.009).

## Discussion

In this study investigating the impact of variation in PTFs of breast cancer on preadjuvant therapy cognitive performance in postmenopausal women with early‐stage breast cancer, we found evidence to support the hypothesis that intertumor heterogeneity of pathologic characteristics may account for variability in pretreatment cognitive function performance. Overall, we report that PTFs related to tumor focality/centricity, tumor location, hormone receptor and HER2 expression, cellular proliferation, as well as Oncotype DX^®^ Breast Cancer Assay Recurrence Score^®^ were significantly (*P* < 0.05) associated with performance for one or more cognitive function composite domains. The most intriguing findings were related to memory performance and HER2 status or HER2 IHC classification score. For all memory composite domains evaluated, diagnosis of a HER2‐positive tumor contributed to poorer performance compared to diagnosis of a HER2‐negative tumor. Likewise, as HER2 IHC classification scores increased, memory performance scores decreased. HER2 is a human epidermal growth factor receptor encoded by the *ERBB2* (*erb‐b2 receptor tyrosine kinase 2*) gene. Within the context of breast cancer, we commonly discuss the oncogenic role of amplification of HER2 and its use as an indicator of more aggressive tumor phenotypes that benefits from targeted therapies (i.e., trastuzumab) 34, 35, 36. Our findings suggest that a more aggressive tumor phenotype, based on HER2 expression, is associated with poorer pretreatment memory performance. These cognition‐related findings are further strengthened when we consider the important and widespread proto‐oncogenic role that *ERBB2* is proposed to play in proper neural development, including formation of aspects of both the central and peripheral nervous systems, and regulation of a variety of adult brain functions 37, 38, 39, 40, 41. Nevertheless, while in line with incidence estimates in the United States, only 8.8% of the study sample was diagnosed with HER2‐positive tumors and, consequently, these findings must be interpreted cautiously and confirmed in additional independent studies 42.

One other interesting finding from the PTF analysis that deserves further discussion was the impact of tumor location, specifically tumor location quadrant or octant, on cognitive functioning. Compared to women diagnosed with a tumor in the upper outer octant, women with a tumor in the lower inner quadrant or octant displayed poorer mental flexibility performance, while women with a tumor in the lower inner quadrant or lower junction octant displayed poorer visual working memory performance. Depending on location classification, previous studies have reported associations between the lower, the inner, and the lower inner breast quadrants and inferior outcomes, including decreased survival and disease recurrence;^43^, ^44^, ^45^, ^46^, ^47^, ^48^ although, conflicting results have also been reported 49. In addition, upper outer quadrant location, the most common location for breast tumors, has been associated with better prognosis compared to other tumor locations 50, 51. These differences in outcomes are hypothesized to occur because of undetected breast cancer spread to the internal mammary lymph nodes 48, 51, 52. While different classifications limit interpretation of results, it is fascinating to note that the tumor locations most strongly related to poorer mental flexibility and visual working memory performance have also been associated with poorer breast cancer outcomes. We further reported differences in verbal and visual working memory performance scores dependent on tumor laterality. The idea that laterality reflects differences in breast cellular biology is not without precedent as left‐right asymmetries in breast cancer have been reported, and findings from the laboratory investigational setting with animal models have revealed dissimilarities in development of mammary glands 53.

In order to facilitate interpretation of the tumor location results, we conducted a post hoc analysis to evaluate how PTFs differed by tumor location octant. This analysis aided in the interpretation of the finding that women with a tumor in the lower junction octant had poorer visual working memory performance when compared to women with tumors in the upper outer octant. A higher than expected percentage of lower junction octant tumors were HER2‐positive compared to the other tumor locations (Fisher's exact test *P* = 0.012). In addition, the lower junction octant displayed higher mean HER2 IHC classification scores compared to the other octants (ANOVA *P* = 0.002). These differences, which relate back to previously discussed associations between memory and HER2 amplification, suggest that PTFs overrepresented in a particular octant may be driving relationships between location and cognitive function rather than the actual location itself. While one published expert opinion suggested that HER2 expression does not vary by anatomic location within the breast, no formal studies have been conducted 54.

Despite this investigation's many strengths including comprehensive evaluation of PTFs reported as part of a surgical pathology report and adjustment for potential covariates/confounders of cognitive function, there are several limitations that should be considered when interpreting the findings. First, as with any retrospective chart review, PTF data were limited to availability in the medical record and recommended testing at the time of diagnosis (e.g., lack of Ki67 proliferative marker evaluation in participants enrolled at the beginning of the parent study). The study sample was comprised of postmenopausal women with hormone receptor positive, early‐stage breast cancer who were primarily Caucasian; the generalizability of study findings to premenopausal women, hormone negative, in situ and more advanced breast cancers, or more diverse populations is unknown. Due to the timing of cognitive assessment (i.e., after primary surgery but prior to initiation of adjuvant therapy) in this study, participants were not blinded to pathology results; the potential impact of participant knowledge of pathology results on cognitive performance is unclear. Additionally, the study sample size limited our ability to analyze and interpret interactions between PTFs or evaluate models with more than one PTF as a predictor.

In conclusion, our results support the hypothesis that variation in pathologic features of breast tumors impact preadjuvant therapy cognitive performance in postmenopausal women with early‐stage breast cancer, with certain factors related to more aggressive tumor phenotypes and inferior prognosis implicated in poorer cognitive performance after surgery to remove the tumor but prior to adjuvant treatment. Of particular interest was the contribution of HER2‐positive breast cancer as well as increasing HER2 IHC classification score to poorer memory performance scores across all memory domains that were assessed. We also found associations between tumor location, tumor focality/centricity, hormone receptor expression, cellular proliferation, and Oncotype DX^®^ Breast Cancer Assay Recurrence Score^®^ and performance for one or more cognitive function composite domains. It will be important to confirm these results in larger, independent studies including more diverse women and cancers as well as studies that include a cognitive assessment prior to primary surgery or core needle biopsy in order to further evaluate the effect of present intertumor heterogeneity on cognitive function and the subsequent cognitive consequences of tumor removal.

## Conflict of Interest

The authors declare that they have no competing interests.

## Supporting information


**Table S1.** PTF and cognitive function robust regression results.
