# Supplementary material for: Associations between pathologic tumor features and preadjuvant therapy cognitive performance in women diagnosed with breast cancer
Source: Cancer Med. 2017 Jan 13;6(2):339–48. doi: 10.1002/cam4.964 (PMC5313647; doi:10.1002/cam4.964)
Supplement: Supplementary file 1 — Table S1. PTF and cognitive function robust regression results. [file CAM4-6-339-s001.pdf]

SUPPLEMENTAL TABLE 1. PTF and Cognitive Function Robust Regression Results

Regression coefficient, p-value	Attention	Concentration	Executive Function	Mental Flexibility	Psychomotor Speed	Verbal Memory	Visual Memory	Visual Working Memory
**AJCC Tumor Stage**	n=321	n=328	n=329	n=328	n=329	n=329	n=329	n=329
Stage IIA	0.116, 0.305	-0.065, 0.550	0.005, 0.949	0.064, 0.472	0.154, 0.075	0.059, 0.485	0.033, 0.550	0.190, 0.033*
Stage IIB	-0.200, 0.262	0.139, 0.425	-0.146, 0.279	.008, 0.953	-0.211, 0.128	-0.157, 0.244	-0.079, 0.363	-0.021, 0.885
Stage IIIA	-0.143, 0.520	-0.207, 0.325	0.034, 0.833	0.179, 0.296	0.087, 0.601	0.068, 0.676	-0.181, 0.088	-0.203, 0.239
Overall p-value	0.354	0.544	0.726	0.694	0.082	0.503	0.218	0.076
Ref: Stage I								
**Tumor Size (cm)**	n=320<br>-0.031, 0.318	n=327<br>-0.032, 0.283	n=328<br>-0.001, 0.955	n=327<br>0.009, 0.717	n=328<br>0.001, 0.951	n=328<br>0.018, 0.418	n=328<br>-0.003, 0.848	n=328<br>0.009, 0.715
**Aggregate Tumor Size (cm)**	n=320<br>-0.027, 0.353	n=327<br>-0.032, 0.249	n=328<br>-0.004, 0.849	n=327<br>0.012, 0.600	n=328<br>-0.010, 0.653	n=328<br>0.004, 0.847	n=328<br>-0.009, 0.544	n=328<br>-0.002, 0.929
**Tumor Classification**	n=321	n=328	n=329	n=328	n=329	n=329	n=329	n=329
T1b	-0.023, 0.890	0.032, 0.842	0.021, 0.866	0.023, 0.858	0.018, 0.893	-0.132, 0.288	0.031, 0.710	0.042, 0.755
T1c	-0.021, 0.896	-0.093. 0.537	0.075, 0.522	0.180, 0.142	0.035,0.774	0.068, 0.557	-0.010, 0.894	0.080, 0.521
T2	-0.013, 0.940	-0.032, 0.848	0.022, 0.868	0.100, 0.464	-0.007, 0.960	-0.051, 0.693	-0.003, 0.970	0.106. 0.446
T3	-0.248, 0.389	-0.238, 0.375	0.051, 0.806	0.133, 0.543	0.115, 0.598	0.140, 0.501	-0.009, 0.946	0.084. 0.708
Overall p-value	0.932	0.723	0.951	0.404	0.977	0.179	0.969	0.943
Ref: T1a								
**Node Positive**<br>*Ref: Negative*	n=318<br>0.058, 0.602	n=324<br>-0.077, 0.473	n-325<br>0.045, 0.587	n=324<br>0.144, 0.101	n=325<br>0.093, 0.280	n=325<br>0.054, 0.514	n=325<br>0.010, 0.859	n=325<br>0.048, 0.595
**Number of Positive Nodes**	n=321<br>0.018, 0.679	n=328<br>-0.024, 0.574	n=329<br>0.004, 0.892	n=328<br>0.057, 0.102	n=329<br>0.040, 0.242	n=329<br>0.008, 0.806	n=329<br>-0.030, 0.172	n=329<br>-0.003, 0.930
**Multi-focal/centric**<br>*Ref: Single*	n=321<br>-0.206, 0.098	n=328<br>-0.056, 0.645	n=329<br>-0.071, 0.450	n=328<br>-0.073, 0.461	n=329<br>-0.092, 0.345	n=329<br>-0.278, 0.003*	n=329<br>-0.084, 0.170	n=329<br>-0.108, 0.280
**Left Breast**<br>*Ref: Right Breast*	n=321<br>0.059, 0.521	n=328<br>-0.109, 0.226	n=329<br>0.019, 0.785	n=328<br>-0.092, 0.204	n=329<br>0.064, 0.366	n=329<br>0.156, 0.025*	n=329<br>-0.034, 0.452	n=329<br>0.163, 0.026*
**Location Octant**	n=315	n=322	n=323	n=322	n=323	n=323	n=323	n=323
Lower Outer	0.357, 0.047*	-0.056, 0.743	0.049, 0.714	-0.064, 0.641	0.023, 0.824	-0.091, 0.492	-0.008, 0.928	0.135, 0.324
Lower Inner	-0.227, 0.253	0.228, 0.238	0.009, 0.955	-0.441, 0.005*	-0.287, 0.062	0.004, 0.978	-0.009, 0.929	-0.166, 0.288
Upper Inner	-0.193, 0.190	0.148, 0.315	0.029, 0.803	-0.204, 0.087	-0.094, 0.420	0.058, 0.612	-0.002, 0.976	-0.120, 0.311
Upper Junction	-0.024, 0.876	0.079, 0.594	0.079, 0.502	-0.135, 0.266	-0.145, 0.221	0.063, 0.588	0.121, 0.110	-0.006, 0.958
Lower Junction	-0.117, 0.579	-0.187, 0.371	0.140, 0.394	-0.270, 0.115	0.101, 0.546	-0.138, 0.401	-0.005, 0.961	-0.387, 0.023*
Outer Junction	0.095, 0.572	-0.139, 0.398	0.188, 0.146	-0.069, 0.603	-0.158, 0.225	0.078, 0.543	0.060, 0.474	0.103, 0.438
Inner Junction	0.506, 0.072	-0.118, 0.672	0.079, 0.718	0.192, 0.400	0.156, 0.483	-0.087, 0.691	0.128, 0.368	0.225, 0.320
Retroareolar	-0.037, 0.877	-0.322, 0.171	0.057, 0.758	-0.259, 0.177	0.129, 0.490	0.055, 0.767	0.228, 0.017*	0.333, 0.081
Overall p-value	0.126	0.490	0.947	0.111	0.374	0.9477	0.331	0.062

Ref: Upper Outer								
**Location Quadrant**<br>Lower Outer<br>Lower Inner<br>Upper Inner<br>Retroareolar<br>Overall p-value<br>*Ref: Upper Outer*	n=315<br>0.201, 0.118<br>-0.176, 0.243<br>-0.076, 0.565<br>-0.029, 0.903<br>0.250	n=322<br>-0.107, 0.389<br>0.049, 0.739<br>0.076, 0.564<br>-0.334, 0.152<br>0.451	n=323<br>0.104, 0.277<br>0.056, 0.623<br>0.023, 0.824<br>0.043, 0.811<br>0.868	n=322<br>-0.040, 0.689<br>-0.335, 0.005*<br>-0.091, 0.394<br>-0.229, 0.225<br>0.068	n=323<br>-0.038, 0.700<br>-0.084, 0.473<br>-0.013, 0.904<br>0.164, 0.374<br>0.805	n=323<br>-0.018, 0.854<br>-0.080, 0.483<br>0.016, 0.870<br>0.039, 0.830<br>0.953	n=323<br>0.006, 0.925<br>-0.036, 0.627<br>-0.011, 0.873<br>0.259, 0.028*<br>0.238	n=323<br>0.123, 0.219<br>-0.267, 0.025*<br>-0.049, 0.644<br>0.331, 0.078<br>0.018*
**Invasive Type**<br>Lobular<br>Ductal & Lobular<br>Overall p-value<br>*Ref: Ductal*	n=320<br>-0.080, 0.595<br>0.505, 0.085<br>0.186	n=327<br>-0.043, 0.772<br>-0.088, 0.760<br>0.920	n=328<br>0.090, 0.432<br>-0.215, 0.335<br>0.441	n=327<br>-0.062, 0.603<br>-0.219, 0.349<br>0.579	n=328<br>-0.034, 0.771<br>-0.209, 0.361<br>0.640	n=328<br>0.107, 0.346<br>-0.003, 0.990<br>0.640	n=328<br>-0.072, 0.335<br>-0.090, 0.537<br>0.536	n=328<br>-0.054, 0.656<br>0.303, 0.203<br>0.388
**Nottingham Score**	n=308<br>-0.032, 0.381	n=314<br>-0.002, 0.958	n=315<br>0.029, 0.291	n=314<br>-0.018, 0.542	n=315<br>0.005, 0.844	n=315<br>0.045, 0.093	n=315<br>0.011, 0.523	n=315<br>0.048, 0.100
**Nottingham Grade**<br>Grade 2<br>Grade 3<br>Overall p-value<br>*Ref: Grade 1*	n=309<br>0.077, 0.467<br>-0.124, 0.392<br>0.312	n=315<br>-0.033, 0.751<br>0.048, 0.733<br>0.817	n=316<br>0.085, 0.288<br>0.132, 0.235<br>0.417	n=315<br>0.000, 0.995<br>-0.070. 0.554<br>0.799	n=316<br>0.066, 0.417<br>-0.009, 0.940<br>0.632	n=316<br>0.111, 0.162<br>0.051, 0.641<br>0.368	n=316<br>0.037, 0.484<br>0.035, 0.635<br>0.771	n=316<br>0.084, 0.329<br>0.202, 0.090<br>0.231
**ER Positive**<br>*Ref: Negative*	n=320<br>0.298, 0.472	n=327<br>-0.290, 0.473	n=328<br>0.211, 0.498	n=327<br>0.391, 0.233	n=328<br>-0.231, 0.471	n=328<br>0.022, 0.945	n=328<br>-0.047, 0.819	n=328<br>-0.269, 0.420
**ER H-Score**	n=303<br>0.001, 0.165	n=310<br>-0.001, 0.409	n=311<br>0.001, 0.397	n=310<br>0.001, 0.301	n=311<br>-0.001, 0.229	n=311<br>0.000, 0.730	n=311<br>0.000, 0.606	n=311<br>0.000, 0.679
**Oncotype DX**<br>**ER Score**	n=100<br>-0.010, 0.904	n=102<br>-0.004, 0.960	n=102<br>0.082, 0.195	n=102<br>0.049, 0.393	n=102<br>0.014, 0.836	n=102<br>-0.013, 0.842	n=102<br>0.074, 0.069	n=102<br>0.047, 0.488
**PR Positive**<br>*Ref: Negative*	n=320<br>-0.129, 0.364	n=327<br>-0.004, 0.975	n=328<br>-0.140, 0.184	n=327<br>0.024, 0.834	n=328<br>0.069, 0.524	n=328<br>-0.256, 0.015*	n=328<br>-0.050, 0.470	n=328<br>-0.048, 0.669
**PR H-Score**	n=302<br>0.000, 0.668	n=309<br>0.000, 0.405	n=310<br>0.000, 0.967	n=309<br>0.000, 0.461	n=310<br>0.000, 0.923	n=310<br>0.000, 0.806	n=310<br>0.000, 0.538	n=310<br>0.000, 0.655
**Oncotype DX**<br>**PR Score**	n=100<br>-0.062, 0.275	n=102<br>0.046. 0.405	n=102<br>-0.029, 0.498	n=102<br>0.010, 0.794	n=102<br>0.031, 0.506	n=102<br>-0.019, 0.650	n=102<br>0.007, 0.782	n=102<br>-0.057, 0.196
**HER2 Positive**<br>*Ref: Negative*	n=310<br>-0.185, 0.283	n=317<br>0.277, 0.087	n=318<br>-0.131, 0.293	n=317<br>-0.065, 0.626	n=318<br>-0.031, 0.803	n=318<br>-0.287, 0.018*	n=318<br>-0.270, 0.001*	n=318<br>-0.490, <0.001*

**HER2 IHC Classification Score**	n=285<br>-0.031, 0.591	n=290<br>0.001, 0.979	n=291<br>-0.018, 0.672	n=290<br>-0.019, 0.679	n=291<br>-0.038, 0.389	n=291<br>-0.072, 0.093	n=291<br>-0.081, 0.003*	n=291<br>-0.170, <0.001*
**Oncotype DX HER2 Score**	n=73<br>-0.106, 0.388	n=74<br>0.105, 0.365	n=74<br>-0.008, 0.925	n=74<br>0.024, 0.753	n=74<br>-0.051, 0.627	n=74<br>-0.041, 0.670	n=74<br>0.011, 0.862	n=74<br>-0.099, 0.288
**LV Invasion**<br>*Ref: No Invasion*	n=315<br>-0.094, 0.420	n=322<br>-0.138, 0.216	n=323<br>0.078, 0.368	n=322<br>0.102, 0.264	n=323<br>-0.076, 0.394	n=323<br>0.095, 0.270	n=323<br>0.018, 0.753	n=323<br>0.059, 0.524
**Ki67 Classification**<br>Moderate<br>High<br>Very High<br>Overall p-value<br>*Ref: Low*	n=168<br>0.001, 0.995<br>0.093, 0.589<br>0.140, 0.507<br>0.872	n=168<br>0.381, 0.009*<br>0.035, 0.829<br>0.254, 0.205<br>0.042*	n=169<br>-0.003, 0.983<br>-0.065, 0.636<br>0.287, 0.090<br>0.266	n=168<br>-0.146, 0.243<br>0.068, 0.635<br>-0.059, 0.740<br>0.481	n=169<br>-0.064, 0.589<br>-0.193, 0.158<br>0.122, 0.463<br>0.325	n=169<br>-0.086, 0.476<br>-0.139, 0.312<br>0.015, 0.927<br>0.711	n=169<br>-0.019, 0.799<br>0.044, 0.602<br>0.031, 0.765<br>0.894	n=169<br>-0.085, 0.475<br>-0.011, 0.936<br>0.073, 0.662<br>0.799
**Ki67 Index**	n=167<br>0.003, 0.359	n=167<br>0.003, 0.342	n=168<br>0.003, 0.135	n=167<br>0.001, 0.712	n=168<br>0.002, 0.343	n=168<br>0.000, 0.906	n=168<br>0.001, 0.722	n=168<br>0.001, 0.540
**Oncotype DX Recurrence Score®**	n=157<br>0.000, 0.957	n=159<br>0.004, 0.483	n=160<br>-0.003, 0.582	n=160<br>-0.010, 0.032*	n=160<br>-0.005, 0.387	n=160<br>0.005, 0.367	n=160<br>-0.003, 0.388	n=160<br>0.009, 0.115
**Magee Equation Recurrence Score**	n=291<br>-0.008, 0.238	n=297<br>0.005, 0.447	n=298<br>0.000, 0.918	n=297<br>-0.002, 0.701	n=298<br>0.002, 0.609	n=298<br>-0.002, 0.615	n=298<br>-0.002, 0.497	n=298<br>-0.002, 0.667

*=p<0.05. Abbreviations: AJCC, American Joint Committee on Cancer; ER, Estrogen Receptor; HER2, Human Epidermal Growth Factor Receptor 2; IHC, Immunohistochemistry; LV, Lymphovascular; Oncotype DX, Genomic Health Inc. Oncotype DX® Breast Cancer Assay; PR, Progesterone Receptor; PTF, Pathologic Tumor Feature; TNM, Tumor, Node, Metastasis Classification of Malignant Tumors; Ref, Reference Group. All regression coefficient estimates and p-values reported from robust multiple linear regression models generated using Huber weighting and biweighting iterations. All models are adjusted for age, estimated verbal intelligence, and levels of depressive symptoms, anxiety, fatigue, and pain.
